# Properties of a non-bioactive fluorescent derivative of differentiation-inducing factor-3, an anti-tumor agent found in *Dictyostelium discoideum*

**DOI:** 10.1242/bio.20146585

**Published:** 2014-03-27

**Authors:** Yuzuru Kubohara, Haruhisa Kikuchi, Yusuke Matsuo, Yoshiteru Oshima, Yoshimi Homma

**Affiliations:** 1 Department of Molecular and Cellular Biology, Institute for Molecular and Cellular Regulation, Gunma University, Maebashi 371-8512, Japan; 2 Laboratory of Natural Product Chemistry, Tohoku University Graduate School of Pharmaceutical Sciences, Sendai 980-8578, Japan; 3 Department of Biomolecular Science, Institute of Biomedical Sciences, Fukushima Medical University School of Medicine, Fukushima 960-1295, Japan

**Keywords:** *Dictyostelium discoideum*, DIF, Anti-tumor agent, Mitochondria, Uncoupler

## Abstract

Differentiation-inducing factor-3 (DIF-3), found in the cellular slime mold *Dictyostelium discoideum*, and its derivatives, such as butoxy-DIF-3 (Bu-DIF-3), are potent anti-tumor agents. To investigate the activity of DIF-like molecules in tumor cells, we recently synthesized a green fluorescent DIF-3 derivative, BODIPY-DIF-3G, and analyzed its bioactivity and cellular localization. In this study, we synthesized a red (orange) fluorescent DIF-3 derivative, BODIPY-DIF-3R, and compared the cellular localization and bioactivities of the two BODIPY-DIF-3s in HeLa human cervical cancer cells. Both fluorescent compounds penetrated the extracellular membrane within 0.5 h and localized mainly to the mitochondria. In formalin-fixed cells, the two BODIPY-DIF-3s also localized to the mitochondria, indicating that the BODIPY-DIF-3s were incorporated into mitochondria independently of the mitochondrial membrane potential. After treatment for 3 days, BODIPY-DIF-3G, but not BODIPY-DIF-3R, induced mitochondrial swelling and suppressed cell proliferation. Interestingly, the swollen mitochondria were stainable with BODIPY-DIF-3G but not with BODIPY-DIF-3R. When added to isolated mitochondria *in vitro*, BODIPY-DIF-3G increased dose-dependently the rate of O_2_ consumption, but BODIPY-DIF-3R did not. These results suggest that the bioactive BODIPY-DIF-3G suppresses cell proliferation, at least in part, by altering mitochondrial activity, whereas the non-bioactive BODIPY-DIF-3R localizes to the mitochondria but does not affect mitochondrial activity or cell proliferation.

## INTRODUCTION

**Fig. 1.**
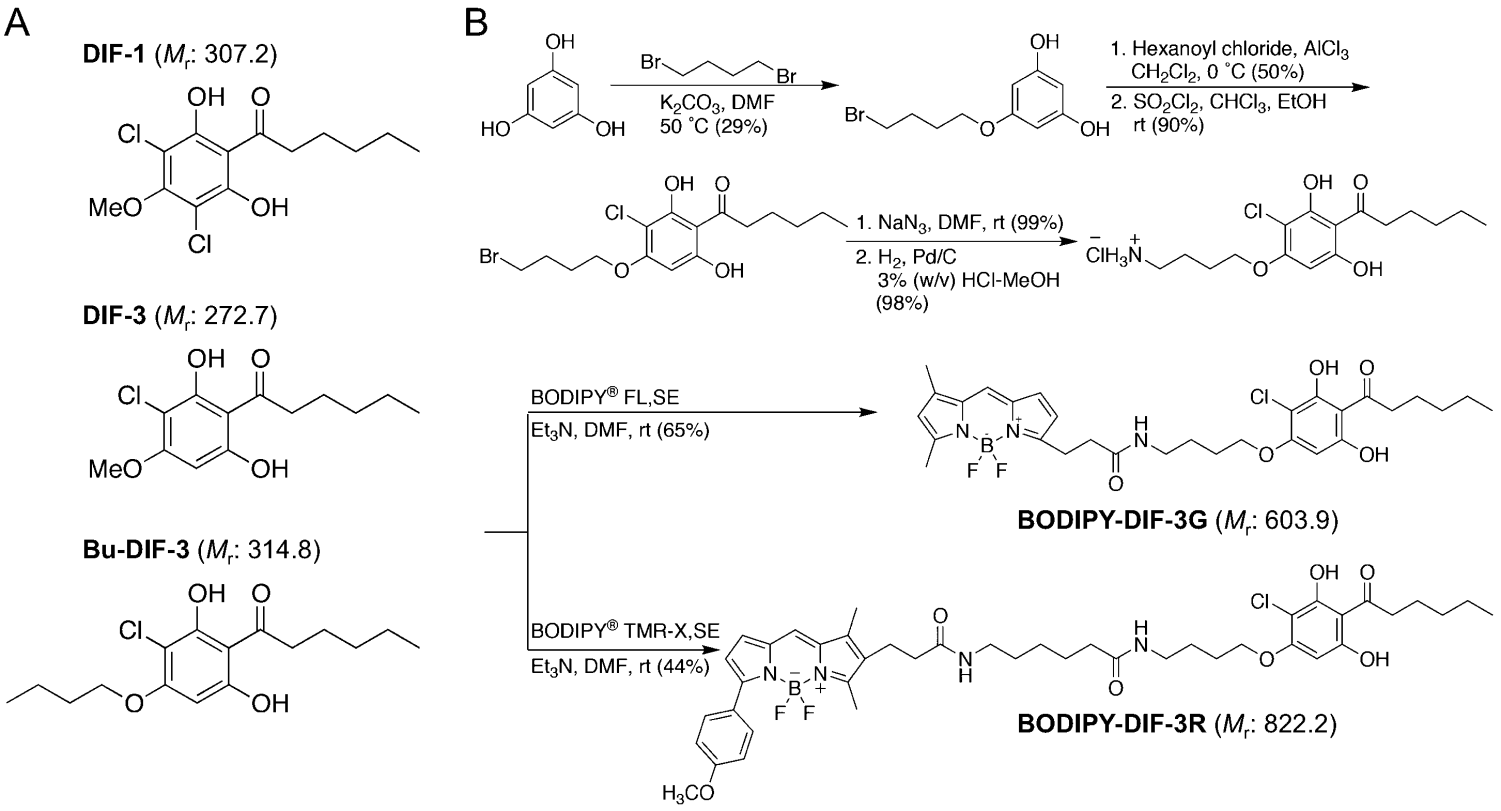
Chemical structures of DIF-1, DIF-3, and Bu-DIF-3, and synthesis of BODIPY-conjugated compounds. (A) DIF-1 [1-(3,5-dichloro-2,6-dihydroxy-4-methoxyphenyl)hexan-1-one] and DIF-3 [1-(3-chloro-2,6-dihydroxy-4-methoxyphenyl)hexan-1-one] are endogenous factors in *D. discoideum*. Bu-DIF-3 (butoxy-DIF-3) [1-(3-chloro-2,6-dihydroxy-4-butoxyphenyl)hexan-1-one] is an artificially designed derivative of DIF-3. The order of anti-proliferative activity has been established as Bu-DIF-3>DIF-3>DIF-1 (Gokan et al., 2005). (B) BODIPY-DIF-3G and BODIPY-DIF-3R were synthesized as described in Materials and Methods. Molecular mass (*M*r) of each compound is indicated in parentheses.

The cellular slime mold *Dictyostelium discoideum* (*D. discoideum*) is a soil microorganism that, at the end of its life cycle, transforms into a multicellular fruiting body consisting of a stalk and spores. Differentiation-inducing factor-1 (DIF-1) (Fig. 1A) is a putative morphogen that regulates cell fate by inducing the differentiation of prestalk cells and suppressing the differentiation of prespore cells (Kay et al., 1989; Kay et al., 1999; Morris et al., 1987). DIF-1 has been shown to function also as a modulator of chemotaxis in *D. discoideum* (Kuwayama and Kubohara, 2009). Differentiation-inducing factor-3 (DIF-3) (Fig. 1A) is the first metabolite formed during DIF-1 degradation and it has virtually no activity in the induction of prestalk cells and the modulation of chemotaxis (Kay et al., 1989; Kay et al., 1999; Kuwayama and Kubohara, 2009; Morris et al., 1988).

DIF-1 and DIF-3 have been shown to suppress tumor cell proliferation as well as induce and promote cell differentiation in de-differentiated tumor cells (Asahi et al., 1995; Jingushi et al., 2012; Jingushi et al., 2013; Kanai et al., 2003; Kubohara et al., 1995; Kubohara, 1997; Kubohara, 1999; Takahashi-Yanaga et al., 2003; Takahashi-Yanaga et al., 2006). Interestingly, DIF-3 is more active than DIF-1 in suppressing cell proliferation and inducing erythroid differentiation of K562 human myelogenous leukemia cells (Akaishi et al., 2004; Kubohara, 1999). We previously found that chemically modified derivatives of DIF-3 such as butoxy-DIF-3 (Bu-DIF-3), which contains a modification at the methoxy group (Fig. 1A), are more potent anti-proliferative agents than DIF-3 in K562 cells (Gokan et al., 2005). Thus, DIF-3 derivatives may be promising anti-cancer drugs. However, the precise mechanisms underlying the function of DIF-like molecules in mammalian cells remain to be elucidated.

To study the cellular localization, function, and target proteins of DIF-3-like molecules in mammalian cells, we recently synthesized a green fluorescent derivative of DIF-3, BODIPY-DIF-3 (designated BODIPY-DIF-3G in this study) (Fig. 1B), and showed that BODIPY-DIF-3G localizes mainly to the mitochondria in HeLa human cervical cancer cells (Kubohara et al., 2013). We also showed that BODIPY-DIF-3G has similar biological activity as DIF-3 and Bu-DIF-3, suppressing cell proliferation in part, by acting as a mitochondrial uncoupler to disrupt mitochondrial function (Kubohara et al., 2013).

In the present study, we synthesized a red (orange) fluorescent derivative of DIF-3, BODIPY-DIF-3R (Fig. 1B), and showed that BODIPY-DIF-3R also localized mainly to the mitochondria. However, unlike BODIPY-DIF-3G and DIF-3, BODIPY-DIF-3R did not suppress HeLa cell proliferation nor induce any change in mitochondrial morphology or function. These results show that bioactive DIF-like molecules suppress cell proliferation at least in part via disturbance of mitochondrial activity, whereas BODIPY-DIF-3R localizes to the mitochondria but is not bioactive. Our results also indicate that the swollen mitochondria are morphologically, biochemically, and thus functionally different, from normal mitochondria and can be distinguished by staining with BODIPY-DIF-3G and BODIPY-DIF-3R.

## RESULTS

### Synthesis of fluorescent derivatives of DIF-3 and their effects on HeLa cell proliferation

We previously synthesized a green fluorescent derivative of DIF-3, BODIPY-DIF-3G (Fig. 1B), and elucidated its cellular localization and function in HeLa cells (Kubohara et al., 2013). Here, we synthesized another reagent for analyzing DIF-like molecules, a red (orange) fluorescent derivative of DIF-3, BODIPY-DIF-3R (Fig. 1B).

**Fig. 2.**
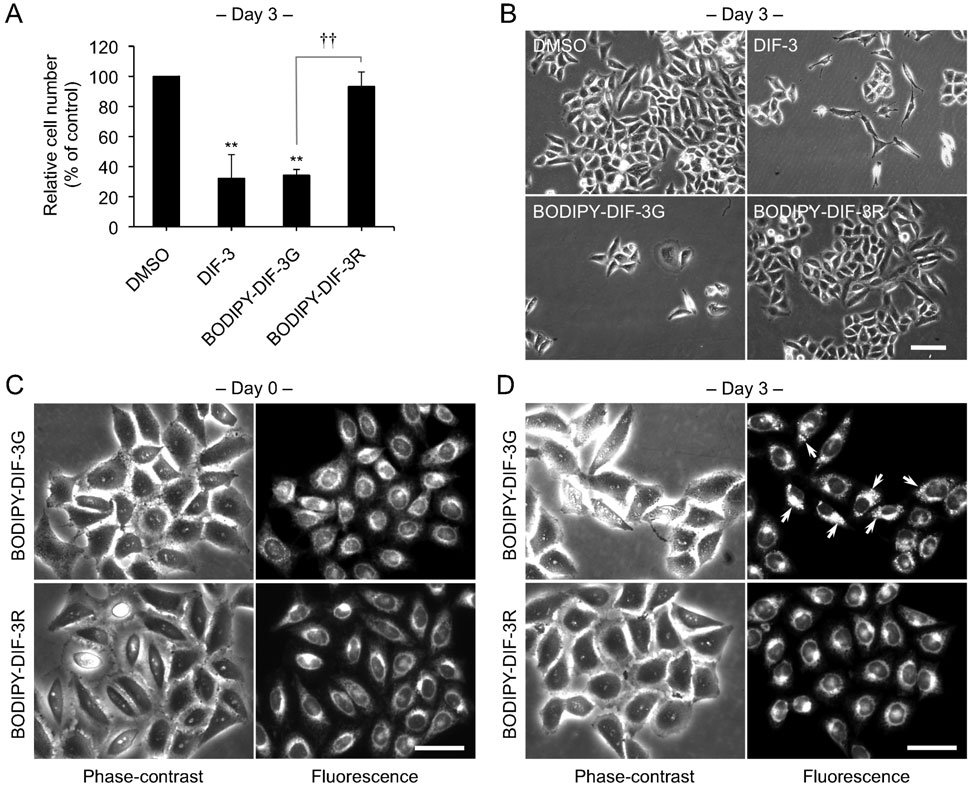
Effects of BODIPY-DIF-3G and BODIPY-DIF-3R on HeLa cell growth, and cellular localization of the BODIPY-conjugated compounds. (A) Cells were incubated for 3 days with 0.2% dimethyl sulfoxide (DMSO; vehicle) or 20 µM of BODIPY-DIF-3G or BODIPY-DIF-3R, and relative cell number was assessed. Mean values and s.d. (bars) of three independent experiments are presented. **P<0.01 versus DMSO control. ^††^*P*<0.01. (B) HeLa cells were incubated for 3 days with 0.2% DMSO or 20 µM of DIF-3, BODIPY-DIF-3G, or BODIPY-DIF-3R, and observed by using phase-contrast microscopy. (C,D) Cells were incubated for 0.5 h (C) or 3 days (D) with BODIPY-DIF-3G (20 µM) or BODIPY-DIF-3R (20 µM), washed free of the additives, and observed by using phase-contrast and fluorescence microscopy. Mitochondrial swelling (arrows) was induced in most of the cells treated with BODIPY-DIF-3G. Scale bars: 100 μm (B), 50 μm (C,D).

We first compared the effects of BODIPY-DIF-3G or BODIPY-DIF-3R on HeLa cell proliferation. As described previously (Kubohara et al., 2013), 20 µM of DIF-3 or BODIPY-DIF-3G markedly suppressed cell proliferation compared to dimethyl sulfoxide (DMSO) control. Unexpectedly, 20 µM BODIPY-DIF-3R had very little effect on cell proliferation (Fig. 2A). Cells remained viable and appeared healthy even after a 3-day incubation period with each of the three compounds (Fig. 2B). Access to both biologically functional and nonfunctional fluorescent DIF-3 derivatives could be powerful tools for studying structure–effect relationships and imaging the cellular localization and function of DIF-like molecules.

### Cellular localization of BODIPY-DIF-3G and BODIPY-DIF-3R in HeLa cells

We next compared cellular localization of BODIPY-DIF-3G and BODIPY-DIF-3R in HeLa cells. Cells incubated for 0.5 h with 20 µM BODIPY-DIF-3G rapidly incorporated the DIF-3 derivative into intracellular organelles that we previously identified as mitochondria (Fig. 2C) (Kubohara et al., 2013). Incubation with BODIPY-DIF-3R under the same conditions resulted in a similar cellular distribution (Fig. 2C). However, while BODIPY-DIF-3G induced mitochondrial swelling (disturbance of the intracellular membrane) after 3 days (Fig. 2D, arrows), BODIPY-DIF-3R scarcely disturbed the intracellular membrane (Fig. 2D).

**Fig. 3.**
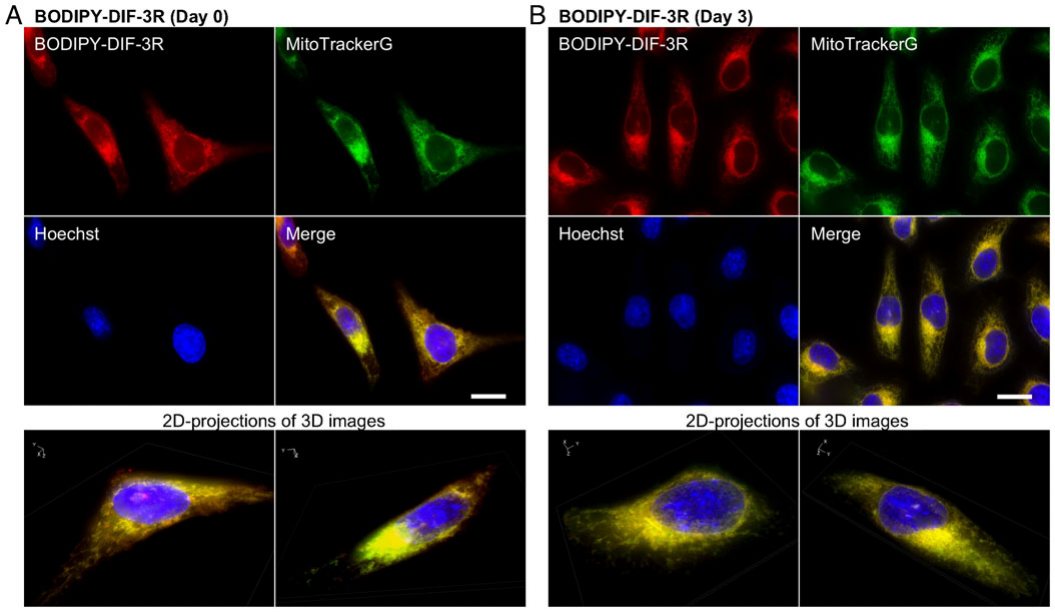
Cellular localization of BODIPY-DIF-3R in HeLa cells. (A) Cells were incubated for 0.5 h with BODIPY-DIF-3R (20 µM), Hoechst (0.1 µg/ml), and MitoTrackerG (0.1 µM), washed free of the additives, and observed by using high-magnification fluorescence microscopy. (B) Cells were incubated for 3 days with BODIPY-DIF-3R (20 µM) and then for 0.5 h with Hoechst (0.1 µg/ml) and MitoTrackerG (0.1 µM). Cells were washed free of the additives and observed by using high-magnification fluorescence microscopy. (A,B) Three-dimensional (3D) images were constructed from z-stacked two-dimensional (2D) images, and two representative 2D-projections of the 3D images are shown. Scale bars: 20 µm.

We then took a more detailed look at the intracellular localization of BODIPY-DIF-3R and BODIPY-DIF-3G under the same conditions in live HeLa cells by using high-magnification fluorescence microscopy and two fluorescent probes for mitochondria, MitoTrackerG and MitoTrackerR. BODIPY-DIF-3R was confirmed to localize mainly in the mitochondria, as shown by its co-localization with MitoTrackerG (Fig. 3A), with no remarkable changes in mitochondrial morphology or cell morphology throughout the 3-day incubation period (Fig. 3B).

**Fig. 4.**
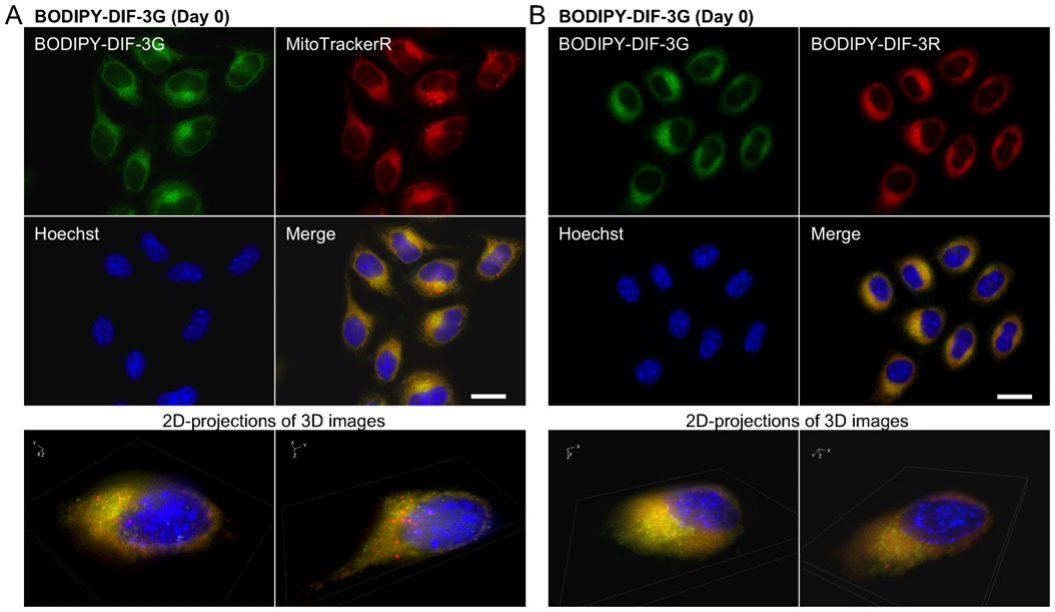
Cellular localization of BODIPY-DIF-3G and BODIPY-DIF-3R in HeLa cells. Cells were incubated for 0.5 h with BODIPY-DIF-3G (20 µM), Hoechst (0.1 µg/ml), and MitoTrackerR (0.1 µM) (A) or BODIPY-DIF-3R (20 µM) (B), washed free of the additives, and observed by using high-magnification fluorescence microscopy. Three-dimensional (3D) images were constructed from z-stacked two-dimensional (2D) images, and two representative 2D-projections of the 3D images are shown. BODIPY-DIF-3G, BODIPY-DIF-3R, and MitoTrackerR co-localized to mitochondria. Scale bars: 20 µm.

**Fig. 5.**
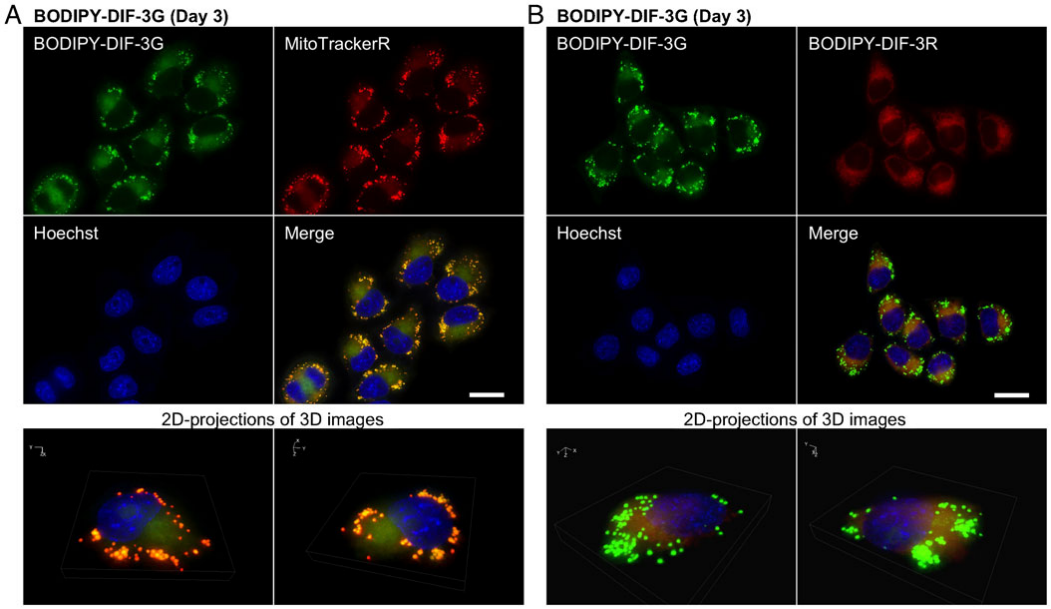
Cellular localization of BODIPY-DIF-3G and BODIPY-DIF-3R in HeLa cells treated for 3 days with BODIPY-DIF-3G. Cells were incubated for 3 days with BODIPY-DIF-3G (20 µM) and then for 0.5 h with Hoechst (0.1 µg/ml) and MitoTrackerR (0.1 µM) (A) or BODIPY-DIF-3R (20 µM) (B). Cells were washed free of the additives and observed by using high-magnification fluorescence microscopy. Three-dimensional (3D) images were constructed from z-stacked two-dimensional (2D) images, and two representative 2D-projections of the 3D images are shown. BODIPY-DIF-3G and MitoTrackerR co-localized to mitochondria, and the swollen mitochondria were stained with BODIPY-DIF-3G and MitoTrackerR, but not BODIPY-DIF-3R. Scale bars: 20 µm.

When cells were incubated for 0.5 h with 20 µM each of BODIPY-DIF-3G and BODIPY-DIF-3R, the two compounds were confirmed to co-localize to the mitochondria (Fig. 4). Incubation for 3 days with 20 µM BODIPY-DIF-3G induced many mitochondria to swell, which were stained well with BODIPY-DIF-3G and MitoTrackerR (Fig. 5A) but were not stained with BODIPY-DIF-3R (Fig. 5B). In contrast, the morphologically normal, but not swollen, mitochondria were stained with both BODIPY-DIF-3G and BODIPY-DIF-3R (Fig. 5B).

### Cellular localization of BODIPY-DIF-3G and BODIPY-DIF-3R in HeLa cells pre-treated with Bu-DIF-3 and carbonyl cyanide *m*-chlorophenyl hydrazine (CCCP)

**Fig. 6.**
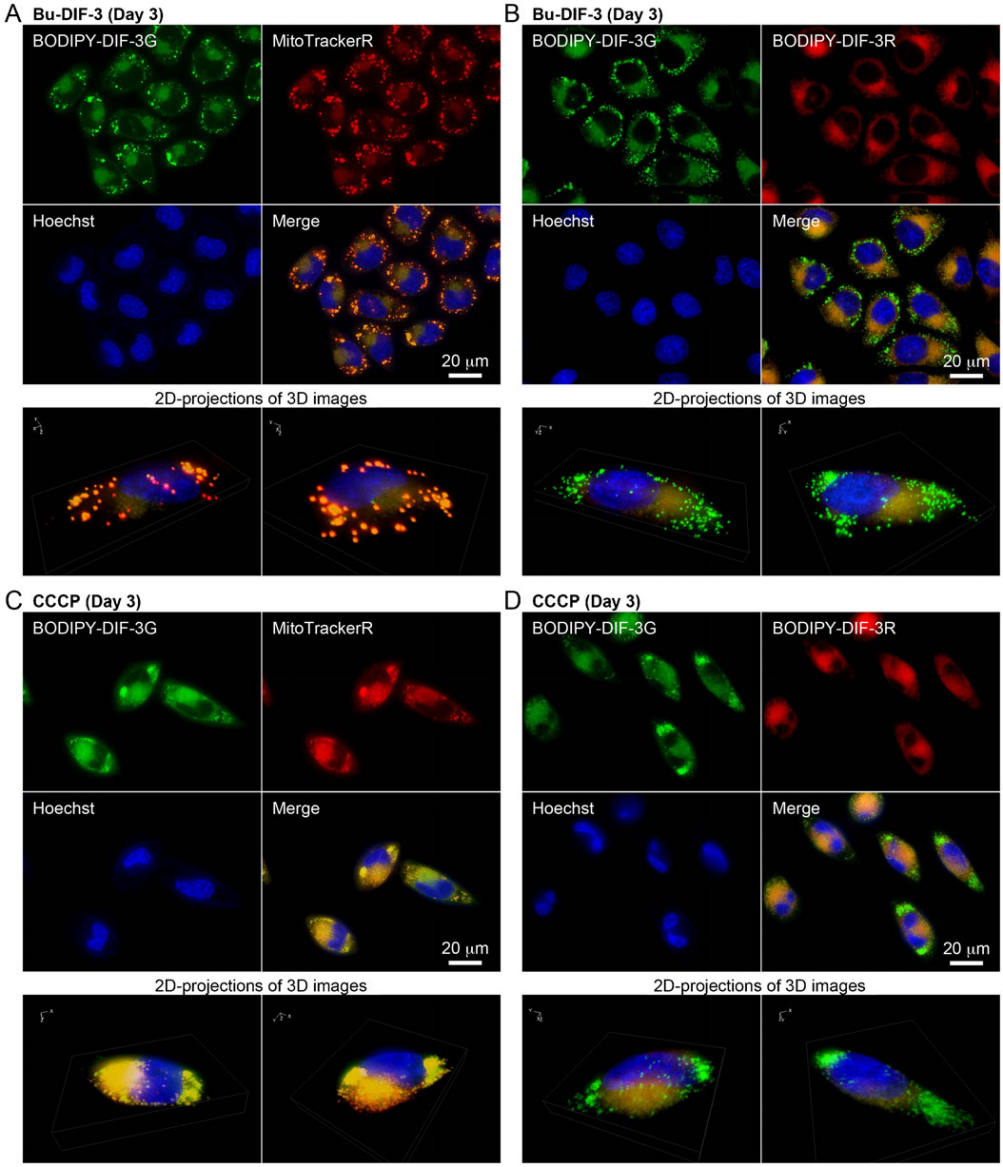
Cellular localization of BODIPY-DIF-3G and BODIPY-DIF-3R in HeLa cells treated for 3 days with Bu-DIF-3 and carbonyl cyanide *m*-chlorophenyl hydrazine (CCCP). Cells were incubated for 3 days with Bu-DIF-3 (5 µM) (A,B) or CCCP (10 µM) (C,D), washed free of the additive, and further incubated for 0.5 h with BODIPY-DIF-3G (20 µM), Hoechst (0.1 µg/ml), and MitoTrackerR (0.1 µM) (A,C) or BODIPY-DIF-3R (20 µM) (B,D). Cells were washed free of the additives and observed by using high-magnification fluorescence microscopy. Three-dimensional (3D) images were constructed from z-stacked two-dimensional (2D) images, and two representative 2D-projections of the 3D images are shown. BODIPY-DIF-3G and MitoTrackerR co-localized to mitochondria, and the swollen mitochondria were stained with BODIPY-DIF-3G and MitoTrackerR, but not BODIPY-DIF-3R. Scale bars: 20 µm.

Bu-DIF-3 is one of the more potent anti-tumor agents among the DIF-like molecules (Gokan et al., 2005), and it has been shown to induce mitochondrial swelling (Kubohara et al., 2013). Therefore, we compared the cellular localization of BODIPY-DIF-3G and BODIPY-DIF-3R in cells treated with 5 µM Bu-DIF-3. After 3 days, mitochondria swelled greatly in all the cells that were stained with BODIPY-DIF-3G and MitoTrackerR (Fig. 6A), but again, BODIPY-DIF-3R stained only the normal, but not swollen mitochondria (Fig. 6B).

CCCP is a mitochondrial uncoupler (proton-specific ionophore) that induces mitochondrial swelling in HeLa cells (Kubohara et al., 2013). We next compared the cellular localization of BODIPY-DIF-3G and BODIPY-DIF-3R in CCCP-treated cells. When cells were incubated for 3 days with 10 µM CCCP, mitochondria swelled greatly in all the cells that were stained with BODIPY-DIF-3G and MitoTrackerR (Fig. 6C), but again, the swollen mitochondria were not stained with BODIPY-DIF-3R (Fig. 6D).

These results show that BODIPY-DIF-3R can penetrate the cell membrane and localize to normal mitochondria, but not to swollen mitochondria induced with the bioactive DIF-3 derivatives BODIPY-DIF-3G or Bu-DIF-3. Functionally, BODIPY-DIF-3R does not induce mitochondrial swelling. In addition, it is likely that the swollen mitochondria are morphologically, biochemically, and thus functionally different, from normal mitochondria and can be distinguished by staining with BODIPY-DIF-3G (or MitoTracker dyes) and BODIPY-DIF-3R.

### Cellular localization of BODIPY-DIF-3G and BODIPY-DIF-3R in formalin-fixed HeLa cells

**Fig. 7.**
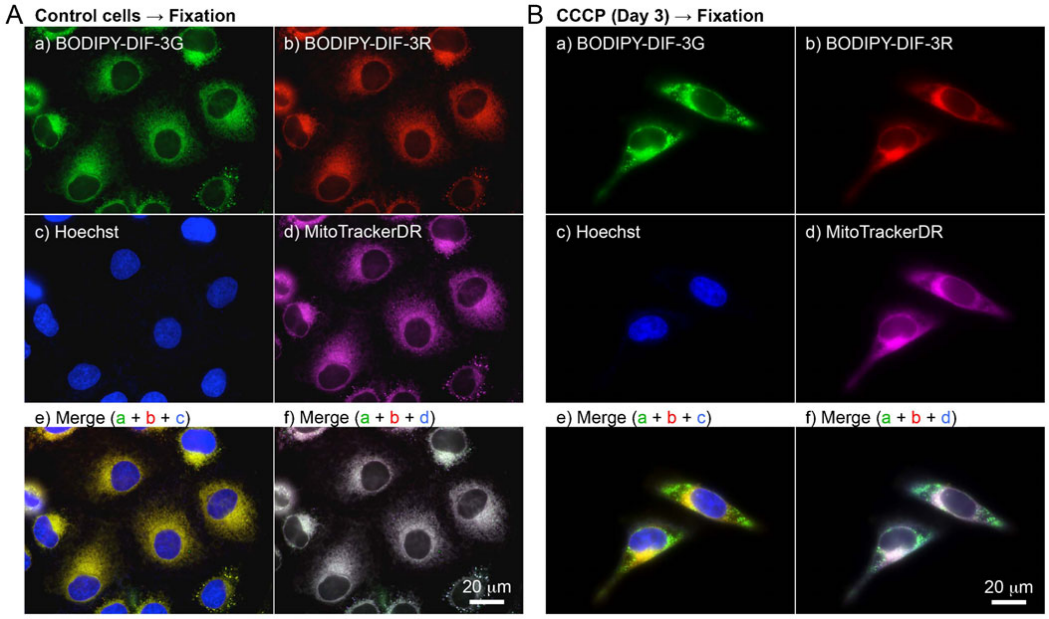
Cellular localization of BODIPY-DIF-3G and BODIPY-DIF-3R in formalin-fixed HeLa cells. Cells were incubated for 3 days without (A) or with (B) CCCP (10 µM) and then incubated for a further 0.5 h with Hoechst (0.1 µg/ml) and MitoTrackerDR (0.2 µM). Cells were washed free of the additives and fixed with 3.7% formalin. The fixed cells were then stained for 0.5 h with BODIPY-DIF-3G (20 µM) and BODIPY-DIF-3R (20 µM), washed free of the additives, and observed by using high-magnification fluorescence microscopy. Merged images (e,f) were constructed with images of cells stained with BODIPY-DIF-3G, BODIPY-DIF-3R, or Hoechst (e) and those stained with BODIPY-DIF-3G, BODIPY-DIF-3R, or MitoTrackerDR (f) with the use of pseudo colors. Scale bars: 20 µm.

We next investigated the cellular localization of BODIPY-DIF-3G and BODIPY-DIF-3R in formalin-fixed HeLa cells (Fig. 7). As described previously (Kubohara et al., 2013), BODIPY-DIF-3G localized to mitochondria that were pre-stained well with MitoTrackerDR, and BODIPY-DIF-3R co-localized to mitochondria in formalin-fixed control cells (Fig. 7A). These results indicate that both BODIPY-DIF-3G and BODIPY-DIF-3R localize to normal-shaped mitochondria independently of the mitochondrial membrane potential and therefore that there may be target molecules of the DIF derivatives within mitochondria. However, CCCP-treated cells (Fig. 7B), in which there appeared to be swollen mitochondria, were hardly stained with MitoTrackerDR. Since accumulation of the probe in active mitochondria is dependent on the mitochondrial membrane potential, the membrane potential must be low in these swollen mitochondria. According to data from the manufacturer, the accumulation of MitoTrackerR in active mitochondria is also dependent on the membrane potential, and since MitoTrackerR was able to stain CCCP-treated swollen mitochondria (Fig. 6C), MitoTrackerR may be a more potent probe than MitoTrackerDR under our experimental conditions. BODIPY-DIF-3G stained both normal-shaped and swollen mitochondria even after fixation, but the swollen mitochondria were only weakly stained with BODIPY-DIF-3R (Fig. 7B), suggesting again that the swollen mitochondria were functionally, biochemically, and/or biophysically different from normal mitochondria.

### Effects of DIFs on mitochondrial O_2_ consumption

**Fig. 8.**
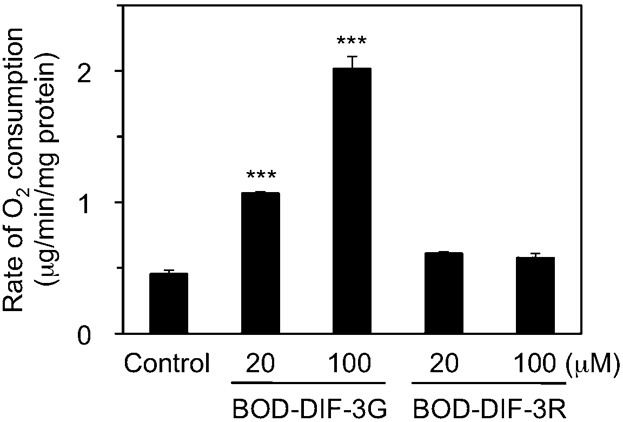
Effects of BODIPY-DIF-3G and BODIPY-DIF-3R on O_2_ consumption of mouse liver mitochondria. Mitochondria were prepared from mouse liver as described in Materials and Methods, and O_2_ concentration in the mitochondria suspension was monitored for several minutes in the presence of 1% DMSO (control), BODIPY-DIF-3G (BOD-DIF-3G; 20 or 100 µM), or BODIPY-DIF-3R (BOD-DIF-3R; 20 or 100 µM). Rate of O_2_ consumption during State 4 respiration (the basal level of the respiration reaction) was calculated, and mean values of three separate measurements and s.d. (bars) are presented. BODIPY-DIF-3R at up to 100 µM scarcely affected the rate of O_2_ consumption, while BODIPY-DIF-3G promoted the rate of O_2_ consumption in a dose-dependent manner. Note that BODIPY-DIF-3R did not affect State 3 respiration induced by the addition of ADP (data not shown). ***P<0.001 versus control.

We have previously shown that the bioactive compounds DIF-3, Bu-DIF-3, and BODIPY-DIF-3G act like mitochondrial uncouplers such as CCCP in that they also increase mitochondrial O_2_ consumption (Kubohara et al., 2013). We examined the effects of BODIPY-DIF-3R on mitochondrial O_2_ consumption in isolated mouse liver mitochondria *in vitro* by using a Clark-type oxygen electrode. As described previously (Kubohara et al., 2013), BODIPY-DIF-3G at 20–100 µM increased basal O_2_ consumption in a dose-dependent manner (Fig. 8). In contrast, the non-bioactive compound, BODIPY-DIF-3R, had no significant effect up to the maximum 100 µM dose. These results suggest that the inability of BODIPY-DIF-3R to suppress cell proliferation is due to its inability to disturb mitochondrial O_2_ consumption.

## DISCUSSION

In addition to *D. discoideum* being studied as a model organism in cell and developmental biology, other cellular slime molds have recently been shown to produce many pharmacologically active compounds (Kikuchi et al., 2004; Kikuchi et al., 2005; Kikuchi et al., 2006; Kikuchi et al., 2010; Kikuchi et al., 2012; Kikuchi et al., 2013). This has led to the suggestion that cellular slime molds are “uncultivated drug resources.” Among the compounds reported so far, the polyketides DIF-1 and DIF-3 (Morris et al., 1987; Morris et al., 1988) are the most promising and well-studied potential anti-tumor drugs (Asahi et al., 1995; Gokan et al., 2005; Kubohara et al., 1995; Kubohara, 1997; Kubohara, 1999). The mechanisms of action of DIF-1 and DIF-3 have been analyzed in several different types of tumor cells but remain to be elucidated (Jingushi et al., 2012; Jingushi et al., 2013; Kanai et al., 2003; Kubohara et al., 1995; Kubohara, 1997; Kubohara, 1999; Kubohara and Hosaka, 1999; Shimizu et al., 2004; Takahashi-Yanaga et al., 2003; Takahashi-Yanaga et al., 2006).

To obtain useful tools for the analyses of the cellular localization and function of DIF-like molecules, we previously synthesized the green fluorescent derivative BODIPY-DIF-3G (Kubohara et al., 2013). In the present study, we described the red (orange) fluorescent derivative BODIPY-DIF-3R. These two different fluorescent DIF-3 derivatives enabled us to simultaneously study their effects on cell proliferation, mitochondrial function, and morphology. Unexpectedly, unlike BODIPY-DIF-3G and DIF-3, BODIPY-DIF-3R did not significantly affect cell proliferation or mitochondrial morphology although it penetrated the cell membrane and localized to normal mitochondria (Fig. 2). In addition, BODIPY-DIF-3G, Bu-DIF-3, and CCCP induced mitochondrial swelling (Fig. 2D, Fig. 5, Fig. 6), whereas BODIPY-DIF-3R did not (Fig. 2D, Fig. 3). Finally, we showed that DIF-3, Bu-DIF-3, and BODIPY-DIF-3G promoted mitochondrial O_2_ consumption in a dose-dependent manner (Kubohara et al., 2013), while BODIPY-DIF-3R did not (Fig. 8). These results suggest that the pharmacologically active DIF-like molecules suppress cell proliferation at least in part by increasing O_2_ consumption rates similar to the action of mitochondrial uncouplers. One reason for the pharmacological inactivity of BODIPY-DIF-3R may be its relatively large molecular mass and/or structure (Fig. 1B). The molecular targets of these DIF-like molecules remain to be identified.

It is important to note that swollen mitochondria induced by BODIPY-DIF-3G, Bu-DIF-3, or CCCP could be stained with BODIPY-DIF-3G, but not with BODIPY-DIF-3R (Figs 5, 6), which was the case even in formalin-fixed cells (Fig. 7), suggesting that swollen mitochondria are functionally, biochemically, and/or biophysically different from normal mitochondria. Elucidation of these differences (e.g. by the use of the two BODIPY-DIF-3 derivatives described in this study) may reveal an unidentified target protein of functional DIF-like molecules in mitochondria.

## MATERIALS AND METHODS

### Reagents and cell culture

MitoTracker® Green FM (designated MitoTrackerG; Ex = 490 nm, Em = 516 nm), MitoTracker® Red CMXRos (MitoTrackerR; Ex = 579, Em = 599 nm), MitoTracker® Deep Red FM (MitoTrackerDR; Ex = 644, Em = 665 nm), BODIPY®FL, SE (succinimidyl ester) (Ex = 505 nm, Em = 513 nm), and BODIPY®TMR-X, SE (Ex = 544 nm, Em = 570 nm) were purchased from Invitrogen (Eugene, OR, USA). Hoechst 33342 (Ex = 352 nm, Em = 461 nm) solution (1 mg/ml H_2_O) and CCCP were obtained from Wako Pure Chemical Industries (Osaka, Japan). DIF-3 and Bu-DIF-3 were synthesized as described previously (Gokan et al., 2005) and stored as 10 mM solutions in DMSO at −20°C.

Human cervical cancer HeLa cells (a kind gift from Dr T. Oda, Gunma University, Japan) (Sekimoto et al., 2010) were maintained *in vitro* at 37°C (5% CO_2_ and 95% air) in DMEM-FBS [Dulbecco's Modified Eagle's Medium (DMEM) containing 4500 mg/l of glucose (Sigma, D5796) supplemented with 75 µg/ml penicillin, 50 µg/ml streptomycin, and 10% (v/v) heat-inactivated fetal bovine serum (FBS)].

### Synthesis of BODIPY-DIF-3G and BODIPY-DIF-3R

BODIPY-DIF-3G was synthesized as described previously (Kubohara et al., 2013), and BODIPY-DIF-3R was synthesized in six reaction steps as described below (Fig. 1B). The synthesized compounds were stored as 10 mM solutions in DMSO at −20°C.

#### Step 1. Synthesis of 5-(4-bromobutoxy)resorcinol

Potassium carbonate (7.17 g, 51.9 mmol) and 1,4-dibromobutane (3.15 ml, 26.4 mmol) were added to a solution of phloroglucinol (4.20 g, 25.9 mmol) in *N*,*N*-dimethylformamide (100 ml) at room temperature. The reaction mixture was stirred for 2 h at 50°C, then diluted with 1.0 M hydrochloric acid (200 ml) and extracted with ethyl acetate (250 ml) three times. The combined organic layer was then washed with water (200 ml) and saturated sodium chloride solution (200 ml), dried over sodium sulfate, and evaporated under reduced pressure. The residue was chromatographed over a silica gel column with a hexane–ethyl acetate (2:1) solvent system to give 5-(4-bromobutoxy)resorcinol (1.94 g, 7.43 mmol).

#### Step 2. Synthesis of 1-(4-(4-bromobutoxy)-2,6-dihydroxyphenyl)hexan-1-one

Hexanoyl chloride (0.720 ml, 5.15 mmol) and aluminum chloride (1.34 g, 10.1 mmol) were added to a solution of 5-(4-bromobutoxy)resorcinol (1.31 g, 5.03 mmol) in dichloromethane (30 ml). The reaction mixture was stirred for 3 h at room temperature, then diluted with water (100 ml) and extracted with ethyl acetate (150 ml) three times. The combined organic layer was washed with saturated sodium bicarbonate solution (150 ml) and saturated sodium chloride solution (150 ml), dried over sodium sulfate, and evaporated under reduced pressure. The residue was chromatographed over a silica gel column with a hexane–ethyl acetate (9:1) solvent system to give 1-(4-(4-bromobutoxy)-2,6-dihydroxyphenyl)hexan-1-one (0.910 mg, 2.53 mmol).

#### Step 3. Synthesis of 1-(4-(4-bromobutoxy)-3-chloro-2,6-dihydroxyphenyl)hexan-1-one

Sulfuryl chloride (342 mg, 2.53 mmol) was added to a solution of 1-(4-(4-bromobutoxy)-2,6-dihydroxyphenyl)hexan-1-one (910 mg, 2.53 mmol) in chloroform–ethanol (49:1) (25 ml). The reaction mixture was stirred for 1 h at room temperature and then evaporated under reduced pressure. The residue was chromatographed over a silica gel column with a hexane–ethyl acetate (9:1) solvent system to give 1-(4-(4-bromobutoxy)-3-chloro-2,6-dihydroxyphenyl)hexan-1-one (899 mg, 2.28 mmol).

#### Step 4. Synthesis of 1-(4-(4-azidobutoxy)-3-chloro-2,6-dihydroxyphenyl)hexan-1-one

Sodium azide (223 mg, 3.42 mmol) was added to a solution of 1-(4-(4-bromobutoxy)-3-chloro-2,6-dihydroxyphenyl)hexan-1-one (337 mg, 0.855 mmol) in *N*,*N*-dimethylformamide (8 ml) at room temperature. The reaction mixture was stirred for 3 h, and then diluted with water (30 ml) and extracted with ethyl acetate (40 ml) three times. The combined organic layer was washed with water (40 ml) and saturated sodium chloride solution (40 ml), dried over sodium sulfate, and evaporated under reduced pressure. The residue was chromatographed over a silica gel column with a hexane–ethyl acetate (4:1) solvent system to give 1-(4-(4-azidobutoxy)-3-chloro-2,6-dihydroxyphenyl)hexan-1-one (302 mg, 0.849 mmol).

#### Step 5. Synthesis of 1-(4-(4-aminobutoxy)-3-chloro-2,6-dihydroxyphenyl)hexan-1-one hydrochloride

Five percent palladium on carbon (2.0 mg) was added to a solution of 1-(4-(4-azidobutoxy)-3-chloro-2,6-dihydroxyphenyl)hexan-1-one (52 mg, 0.145 mmol) in 3% (w/v) hydrochloride methanol solution (3 ml) at room temperature. The reaction mixture was stirred for 1 h under a hydrogen atmosphere and then filtered through a Celite pad. The Celite pad was washed with methanol and the filtrate was evaporated under reduced pressure. The residue was chromatographed over a silica gel column with a chloroform–methanol (4:1) solvent system to give 1-(4-(4-aminobutoxy)-3-chloro-2,6-dihydroxyphenyl)hexan-1-one hydrochloride (52 mg, 0.142 mmol).

#### Step 6. Synthesis of BODIPY-DIF-3R

1-(4-(4-Aminobutoxy)-3-chloro-2,6-dihydroxyphenyl)hexan-1-one hydrochloride (7.9 mg, 21.5 µmol) and triethylamine (20 µl) were added to a solution of BODIPY®TMR-X, SE (2.6 mg, 4.3 µmol) in tetrahydrofuran (1 ml) at room temperature in the dark. The reaction mixture was stirred for 5 h, and then diluted with 0.2 M hydrochloric acid (5 ml) and extracted with ethyl acetate (10 ml) three times. The residue was subjected to recycling preparative HPLC (column, JAIGEL-GS-310, φ 20 mm × 500 mm, Japan Analytical Industry Co., Ltd. Tokyo, Japan; solvent, ethyl acetate) to give BODIPY-DIF-3R (1.5 mg, 1.8 µmol). Analytical data for BODIPY-DIF-3R: ^1^H NMR (400 MHz, CDCl_3_) δ 7.87 (1H, d, *J* = 8.9 Hz), 7.09 (1H, s), 6.97 (1H, d, *J* = 8.9 Hz), 6.95 (1H, d, *J* = 4.0 Hz), 6.55 (1H, d, *J* = 4.0 Hz), 5.61–5.72 (2H, m), 4.02 (2H, t, *J* = 6.5 Hz), 3.85 (3H, s), 3.25 (2H, q, *J* = 7.2 Hz), 3.19 (2H, q, *J* = 7.4 Hz), 3.08 (2H, t, *J* = 7.5 Hz), 2.77 (2H, t, *J* = 7.2 Hz), 2.77 (2H, t, *J* = 7.1 Hz), 2.53 (3H, s), 2.30 (2H, t, *J* = 7.1 Hz), 2.21 (3H, s), 2.05 (2H, t, *J* = 7.1 Hz), 1.77 (2H, quint, *J* = 6.5 Hz), 1.56–1.69 (4H, m), 1.20–1.45 (10H, m), 0.91 (3H, t, *J* = 7.0 Hz); HRFABMS *m/z* 803.3778 [M-F]^+^ (803.3758 calculated for C_43_H_54_N_4_O_7_B^35^ClF).

### Cell proliferation assay

HeLa cells were cultured in 12-well plates for 3 days at 2.5–5×10^3^ cells/well in 1 ml of DMEM-FBS containing 0.2% DMSO or 20 µM of DIF-3, BODIPY-DIF-3G, or BODIPY-DIF-3R. Cells were treated with trypsin and the detached cells were used for direct cell number count using a hematometer, and relative cell number was calculated.

### Phase-contrast and fluorescence microscopy

For low-magnification observation, HeLa cells were incubated for 0.5 h or 3 days with 2 ml of DMEM-FBS containing 0.2% DMSO or 20 µM of DIF-3, BODIPY-DIF-3G, or BODIPY-DIF-3R in 35-mm Falcon tissue culture dishes (Becton Dickinson Labware, Franklin Lakes, NJ, USA). The cells were washed twice with PBS(−) and submerged in 2 ml HEPES buffer (0.1% bovine serum albumin, 137.5 mM NaCl, 5 mM KCl, 2.5 mM CaCl_2_, 0.8 mM MgCl_2_, 5.5 mM glucose, 0.6 mM NaHCO_3_, 20 mM HEPES-NaOH; pH 7.4). The cells were observed by using a Leica DM IRB fluorescent microscope (Wetzlar, Germany), and digitized images were analyzed by using the Leica Application Suite (version 3.3.0).

For high-magnification observation and multi-color imaging, cells were incubated for 0.5 h or 3 days with 2 ml DMEM-FBS containing additives in 35-mm tissue culture treated plastic 81156 μ-dishes (ibidi, Martinsried, Germany). The cells were washed twice with 2 ml of PBS(−), submerged in 2 ml of HEPES buffer (pH 7.4) and observed by using a Keyence BZ-9000 fluorescence microscope (Osaka, Japan) equipped with an oil immersion 100× lens (CFI Plan Apo VC100XH) and multi-filters that distinguish up to four fluorescent probes simultaneously. Digitized images of z-stack sections were taken at 0.4-µm intervals, which were then treated (haze-reduced) with the Keyence BZ analyzer software (for deconvolution fluorescence imaging) and compiled into three-dimensional (3D) images; when 3D images were constructed, nonlinear adjustment was performed to obtain clear (high contrast) images without haze. All color images are presented in pseudo colors.

### Observation of formalin-fixed cells

HeLa cells were incubated for 3 days with 2 ml of DMEM-FBS in 35-mm tissue culture plastic 81156 μ-dishes in the presence or absence of CCCP (10 µM), and then incubated for a further 0.5 h with Hoechst 33342 (0.1 µg/ml) and MitoTrackerDR (0.2 µM), which are dyes that are retained in nuclei and mitochondria, respectively, after fixation. Cells were washed twice with PBS(−) and fixed for 20 min at room temperature with 2 ml of 3.7% (v/v) formaldehyde in PBS(−). After being washed 3 times with PBS(−), the fixed cells were incubated for 0.5 h with 20 µM BODIPY-DIF-3G and BODIPY-DIF-3R in 2 ml of DMEM-FBS, washed 3 times with PBS(−), and observed and analyzed with a Keyence BZ-9000 fluorescence microscope and BZ analyzer software as described above.

### Preparation of mitochondria-enriched cell fraction and measurement of O_2_ consumption

Mitochondria were isolated from mouse liver (ICR, 7–10-week-old females) by differential centrifugation as described previously (Kabuyama et al., 2010; Kubohara et al., 2013). Mitochondrial O_2_ consumption was determined by using a Clark-type oxygen electrode (Strathkelvin Instruments Ltd., North Lanarkshire, Scotland) as described (Gottlieb et al., 2002; Wegrzyn et al., 2009). The mitochondria-enriched fraction was incubated in O_2_ measurement buffer (225 mM mannitol, 75 mM sucrose, 10 mM KCl, 0.1 mM EDTA, 3 mM phosphate, 5 mM succinate, 5 mM glutamate, 20 mM Tris-HCl; pH 7.4) in the presence of 1% DMSO, BODIPY-DIF-3G (20 or 100 µM), or BODIPY-DIF-3R (20 or 100 µM) at 30°C. After recording the mitochondrial respiration State 4 reaction, an aliquot of ADP was added to a final concentration of 200 µM to induce State 3 respiration. However, since DIF-related molecules have been shown to affect State 4 respiration (Kubohara et al., 2013), the rate of O_2_ consumption during State 4 was calculated and compared in this study.

### Statistics

Significance was assessed by unpaired (two-tailed) Student's *t*-test. Values were considered significantly different when the *P* value was less than 0.05.
